# Walking ability of individuals fitted with transfemoral bone-anchored prostheses: A comparative study of gait parameters

**DOI:** 10.1177/02692155231183779

**Published:** 2023-06-23

**Authors:** Simone Ranaldi, Alexandre Naaim, Cristiano De Marchis, Thomas Robert, Raphael Dumas, Silvia Conforto, Laurent Frossard

**Affiliations:** 1 BioLab³ – Engineering Department, 19012 Roma TRE University, Rome, Lazio, Italy; 2 Univ Lyon, Univ Gustave Eiffel, Univ Claude Bernard Lyon 1, LBMC UMR T_9406, Lyon, France; 3 Department of Engineering, 18980 University of Messina, Messina, Sicilia, Italy; 4 Griffith Centre of Biomedical and Rehabilitation Engineering (GCORE), 5723 Griffith University, Southport, QLD, Australia; 5 YourResearchProject, Red Hill, QLD, Australia

**Keywords:** Amputation, Artificial Limbs, bone-anchored prosthesis, gait parameters, prosthesis

## Abstract

**Objective:**

This study presents the walking abilities of participants fitted with transfemoral bone-anchored prostheses using a total of 14 gait parameters.

**Design:**

Two-centre retrospective cross-sectional comparative study.

**Setting:**

Research facilities equipped with tridimensional motion capture systems.

**Participants:**

Two control arms included eight able-bodied participants arm (54 ± 9 years, 1.75 ± 0.07 m, 76 ± 7 kg) and nine participants fitted with transfemoral socket-suspended prostheses arm (59 ± 9 years, 1.73 ± 0.07 m, 80 ± 16 kg). The intervention arm included nine participants fitted with transfemoral bone-anchored prostheses arm (51 ± 13 years, 1.78 ± 0.09 m, 87.3 ± 16.1 kg).

**Intervention:**

Fitting of transfemoral bone-anchored prostheses.

**Main measures:**

Comparisons were performed for two spatio-temporal, three spatial and nine temporal gait parameters.

**Results:**

The cadence and speed of walking were 107 ± 6 steps/min and 1.23 ± 0.19 m/s for the able-bodied participants arm, 88 ± 7 steps/min and 0.87 ± 0.17 m/s for the socket-suspended prosthesis arm, and 96 ± 6 steps/min and 1.03 ± 0.17 m/s for bone-anchored prosthesis arm, respectively. Able-bodied participants and bone-anchored prosthesis arms were comparable in age, height, and body mass index as well as cadence and speed of walking, but the able-bodied participant arm showed a swing phase 31% shorter. Bone-anchored and socket-suspended prostheses arms were comparable for age, height, mass, and body mass index as well as cadence and speed of walking, but the bone-anchored prosthesis arm showed a step width and duration of double support in seconds 65% and 41% shorter, respectively.

**Conclusions:**

Bone-anchored and socket-suspended prostheses restored equally well the gait parameters at a self-selected speed. This benchmark data provides new insights into the walking ability of individuals using transfemoral bionics bone-anchored prostheses.

## Introduction

**Figure 1.**
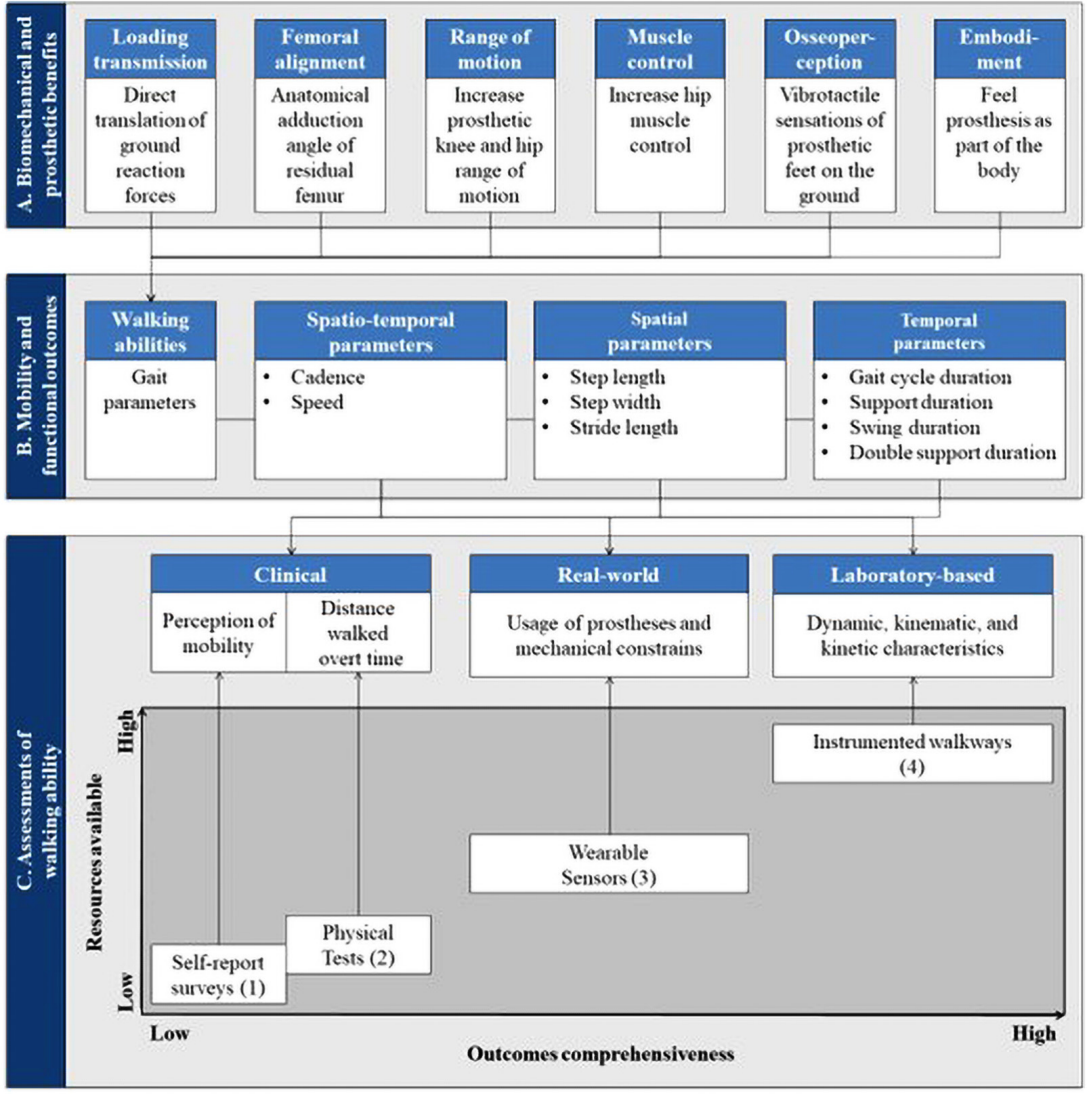
Overview of links between biomechanical and prosthetic benefits of the transfemoral osseointegrated implant (A), mobility and functional outcomes of individuals fitted with a transfemoral bone-anchored prosthesis (B), and assessments of gait parameters (C) resulting in a trade-off between the resources available (e.g. time, cost, equipment and space) and the outcomes comprehensiveness (e.g. standard, range, realism and accuracy). (1) 36-Item Short Form Survey, Questionnaire for Persons with a Transfemoral Amputation, Amputee Mobility Predictor Assessment Tool, (2) Timed Up and Go Test, 6-min walk test Six-Minute Walk Test, 10-min walk test Six-Minute Walk Test, (3) iPecLab, and (4) 3D motion capture, force-plates.

The biomechanical and prosthetic benefits of lower limb bone-anchored prostheses attached to osseointegrated implants are likely to increase mobility (Figure 1).^1–7^ Functional outcomes of transfemoral bone-anchored prostheses are commonly reported using basic cadence and speed of walking.^8–10^ As a result, our understanding of the effects of this type of prostheses on gait parameters is limited.^11^ There is a need for the comparisons of a series of gait parameters produced by individuals with transfemoral bone-anchored and socket-suspended prostheses as well as able-bodied participants (Supplemental Table S1). The purpose of this study is to compare gait spatio-temporal parameters between these three populations. The objectives were to present:

1. Descriptive statistics and variability of two spatio-temporal, three spatial and nine temporal gait parameters for cohorts of eight able-bodied participants (ABD arm), nine individuals with transfemoral socket prosthesis (SSP arm), and nine individuals with transfemoral bone-anchored prosthesis (BAP arm).
2. Possible confounding effects of age, height, mass, and body mass index on each of these 14 gait parameters, considering 56 correlations per cohort giving 168 correlations in total.
3. Differences for 14 gait parameters between ABD and SSP, ABD and BAP, as well as BAP and SSP arms, looking at 42 percentages of difference and statistical differences.

## Methods

This study was designed as a two-centre retrospective cross-sectional comparative study performed in Istituto nazionale Assicurazione Infortuni sul Lavoro (INAIL) Prosthetic Centre, Roma, Italy (Centre A) and Department of Prosthetics and Orthotics of Sahlgrenska, University Hospital, Goteborg, Sweden (Center B), including two control arms with able-bodied participants (ABD arm) and individuals with transfemoral socket-suspended prostheses (SSP arm) and an intervention arm with participants fitted transfemoral bone-anchored prostheses (BAP arm) (Supplemental Figure S1). The methodological aspects associated with recording, extraction and analysis of the gait parameters for each Experiment 1 (ABD arm), 2 (SSP arm) and 3 (BAP arm) are detailed here. Complementary information was outlined in previous publications.^12–15^

Methodological information related to participants, recruitment, prosthetic components, measurements, equipment, and conditions for each arm is detailed in Table 1.

**Table 1.** Overview of the experiments in the three arms of the study including ABD as well as individuals with transfemoral amputation fitted with SSP and BAP arms.

	Control arms		Intervention arm
Study design			
Experiment	1	2	3
Centre	Istituto nazionale Assicurazione Infortuni sul Lavoro (INAIL) Prosthetic Centre, Roma, Italy (Centre A)		Department of Prosthetics and Orthotics of Sahlgrenska, University Hospital, Goteborg, Sweden (Centre B)
Population	ABD	SSP	BAP [1]
Comparisons			
1	X	X	
2	X		X
3		X	X
Participants			
Number	8	9	9 [2]
Male	8	9	8
Female	–	–	1
Recruitment			
Timeline	May 2017 Dec 2018	Sept 2016 Dec 2018	Aug 2002
Selection criteria			
Exclusion	- No history of neurological pathologies	- Non-prosthetic user	- Self-reported pain level greater than four out of 10 - Fall within the last 8 weeks before assessment - Signs of infection 2 weeks prior testing session
Inclusion		- Fully rehabilitated - Able to walk 200 m independently	- Fully rehabilitated - Able to walk 200 m independently - Minimum of 6 cm clearance between percutaneous part and knee
Prosthetic components		[3]	[4] [5]
Non-Microprocessor-controlled knee		7 [6]	7 [7]
Microprocessor-controlled knee		2 [8]	2 [9]
Energy storing and return ankle/foot		9 [10]	9 [11]
Apparatus			
Length of walkway	9 m		8 m
Motion capture			
Model and brand	BTS Bioengineering SMART-DX 6000 optoelectronic system (BTS Bioengineering, Italy)		ProReflex 240 3D Motion Capture Unit (Qualisys, Sweden)
Number of cameras	7		6
Force-plates			
Model and brand	Kistler		Kistler
Number of force-plates	2		2
Marker sets			
Set	Davis’ set		Customised marker set
Number of markers	22		15 [12]
Conditions			
Number of trials	10 [12]		5 [13]
Number of gait cycles	42	33	86

ABD: able-bodied participants; SSP: socket-suspended prosthesis; BAP: bone-anchored prosthesis; BMI: body mass index.

[1] Cohort fitted with screw-type implant (OPRA, Integrum AB); [2] cohort representing approximately 15% of the population of individuals with transfemoral amputation fitted with BAP worldwide at the time of the recording; [3] participants used their own usual footwear as well as knee and ankle/foot components; [4] participants were fitted with instrumented prosthesis included a transducer and their own footwear as well as usual knee and ankle/foot components; [5] the alignment with the instrumented prosthesis was mainly preserved for all participants except one when the knee was dropped by a few centimetres to fit the transducer; [6] mechanical knees (*N* = 7); [7] single-axis GaitMaster (*N* = 1), polycentric total knee 1900 (*N* = 5), single-axis adaptive (*N* = 1); [8] C-Leg (*N* = 1), Genium X3 (*N* = 1); [9] C-Leg (*N* = 2); [10] Variflex (*N* = 9); [11] TruStep (*N* = 3), total concept (*N* = 1), carbon copy (*N* = 2), C-Walk (*N* = 2), flex foot (*N* = 1), unknown (*N* = 1); [12] customised marker set adjusted as presented in Dumas et al. including 13 markers on lower limbs; [13] additional trials were recorded, when needed, to achieve at least three complete landings of both feet on the hidden force plates; [14] participants landed their prosthetic and sound feet on the first and second force-plate so that the direction on the walkway was inverted depending on the side of amputation so that the prosthetic limb always stepped on the force plate first.

For all three experiments, the participants were:

- Recruited by local prosthetists based on particular selection criteria presented previously.^12^ In all cases, participants were active with an overall fairly high ambulatory capacity. No exclusion criteria were applied for gender, ethnicity, age, weight and height or level of activity.
- Required to sign a written ethical consent form approved by the research organization's human ethics committee at the time of the study.
- Asked to perform straight-level walking at a self-selected comfortable speed.

Participants in the SSP arm (Control) were matched 1:1 to participants in the BAP arm (Intervention) based primarily on age, height, mass and body mass index. The participants in this control arm who had the greatest number of matching criteria and matching values closest to those identified for matched participants in the intervention arm were selected as the matching control participants. The ABD arm (Control) was inherited from previous studies and considered without a matching process with BAP and SSP arms.

The 14 gait parameters of the prosthetic limb for SSP and BAP arms and single limb for the ABD arm were processed in a specifically written Matlab program (The MathWorks Inc., Massachusetts, United States of America) following a seven-step process detailed in Supplemental Table S2 (i.e. import raw data, identify gait events, harmonize coordinate systems, calculate gait parameters, normalize datasets, aggregate datasets, determine confounding effects of demographics).^13^

In step 6, the variability of a dataset across all trials and participants was determined using the percentage of variation (i.e. percentage of variation absolute = [(standard deviation/mean) × 100]). We considered that the percentage of variation inferior or superior to 20% indicated a low and high variability, respectively.^12,13^

The demographics and gait parameters datasets were represented using boxplots showing median value, interquartile range and full range without outliers (i.e. range computed after having excluded them) that were 1.5 folds below the first or above the third interquartile range.

The effect of potential confounders (i.e. age, height, mass, and body mass index) on 14 gait parameters was established using the coefficient of determination (*r*^2^), based on the Pearson correlation coefficient (*r*), which we categorised as very weak (*r*^2^ ≤ 0.25), weak (0.25 < *r*^2^ ≤ 0.50), moderate (0.50 < *r*^2^ ≤ 0.75) and substantial (*r*^2^ > 0.75).

Differences in 14 gait parameters between ABD and SSP (Comparison 1), ABD and BAP (Comparison 2), as well as BAP and SSP (Comparison 3) arms giving a total of 42 comparisons, were expressed in units, and the percentage of difference (e.g. [[ABD-SSP]/ABD)*100]). For each comparison, a statistical analysis was carried out using a Kruskal-Wallis test with the level of significance set to α = 0.05/3 = 0.017 (Bonferroni correction for three group comparisons).

Finally, the step length and cadence were used to estimate the performances for the equivalent of a 6-min walk test in order to facilitate comparison with literature and inform generalization of the outcomes. The speed of walking and distance walked were extracted or recalculated for three comparable studies that presented either a 6 or 10-min walk test considering that the speed of walking was sustained for both tests.^8–10^ We considered averaged outcomes of these three published studies.

## Results

**Figure 2.**
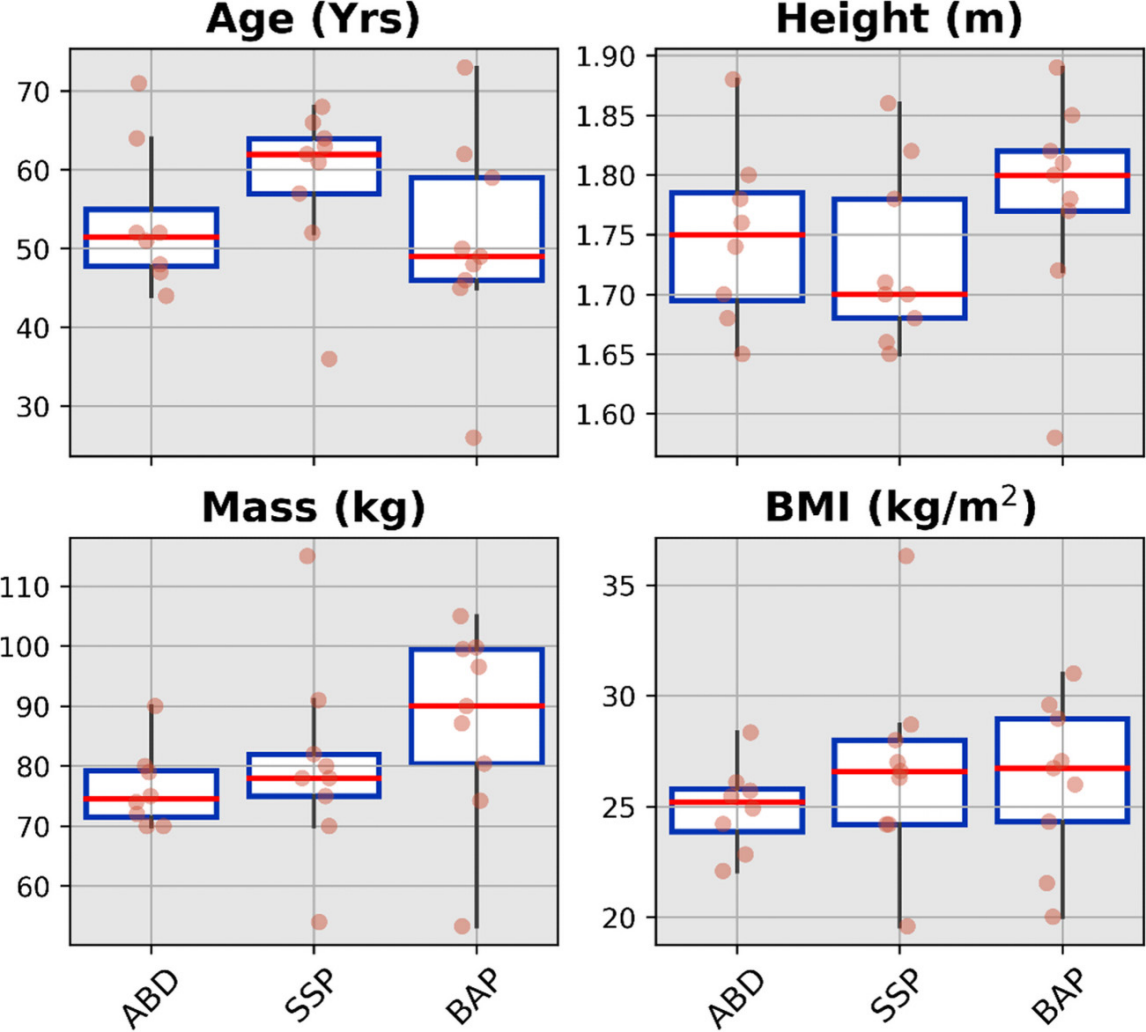
Boxplots showing median value, interquartile range and full range without outliers of the demographics for ABD (54 ± 9 years, 1.75 ± 0.07 m, 76 ± 7 kg, 25.0 ± 1.0 kg/m^2^) as well as individuals with transfemoral amputation fitted with SSP (59 ± 9 years, 1.73 ± 0.07 m, 80 ± 16 kg, 26.8 ± 4.5 kg/m^2^) and BAP (51 ± 13 years, 1.78 ± 0.09 m, 87.3 ± 16.1 kg, 26.1 ± 3.7 kg/m^2^) prostheses arms. ABD: able-bodied participants; SSP: socket-suspended prosthesis; BAP: bone-anchored prosthesis.

The mean and standard deviation as well as median value, interquartile range and full range without outliers of the age, height, mass and body mass index for each cohort were presented in Figure 2 as well as Supplemental Table S3.

Comparisons of the demographics between the three cohorts outlined in Table 2 showed no significant differences.

**Table 2.** Comparisons of the demographics between participants in ABD as well as individuals with transfemoral amputation fitted with SSP and BAP arms showing no significant difference (*p* < 0.017).

	ABD arm versus SSP arm		ABD arm versus BAP arm		BAP arm versus SSP arm	
	Mean ± SD	*p*-value	Mean ± SD	*p*-value	Mean ± SD	*p*-value
Age (yrs)	−5.15 ± 4.60	0.210	2.74 ± 8.26	0.680	−7.89 ± 5.44	0.090
Height (m)	0.02 ± 0.04	0.620	−0.03 ± 0.04	0.230	0.05 ± 0.04	0.150
Mass (kg)	−4.08 ± 5.96	0.410	−11.07 ± 5.88	0.040	6.99 ± 7.68	0.250
Body mass index (kg/m^2^)	−1.810 ± 1.650	0.270	−1.180 ± 1.350	0.320	−0.630 ± 1.930	0.840

SD: one standard deviation; ABD: able-bodied participants; SSP: socket-suspended prosthesis; BAP: bone-anchored prosthesis; BMI: body mass index.

A total of 161 gait cycles were analysed, including 42 (26%), 33 (21%) and 86 (53%) cycles collected during Experiments 1, 2 and 3, respectively.

**Figure 3.**
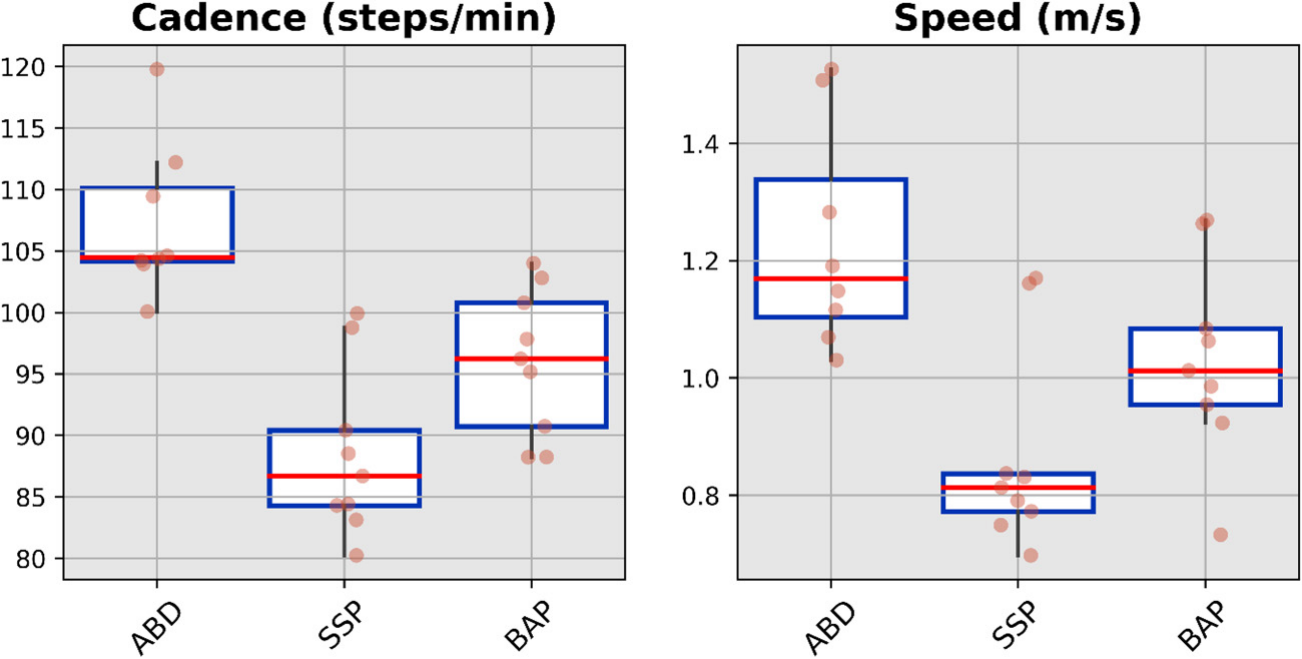
Boxplots showing median value, interquartile range and full-range without outliers of the spatio-temporal parameters for able-bodied participants (ABD) as well as individuals with transfemoral amputation fitted with socket-suspended (SSP) and bone-anchored (BAP) prostheses arms.

**Figure 4.**
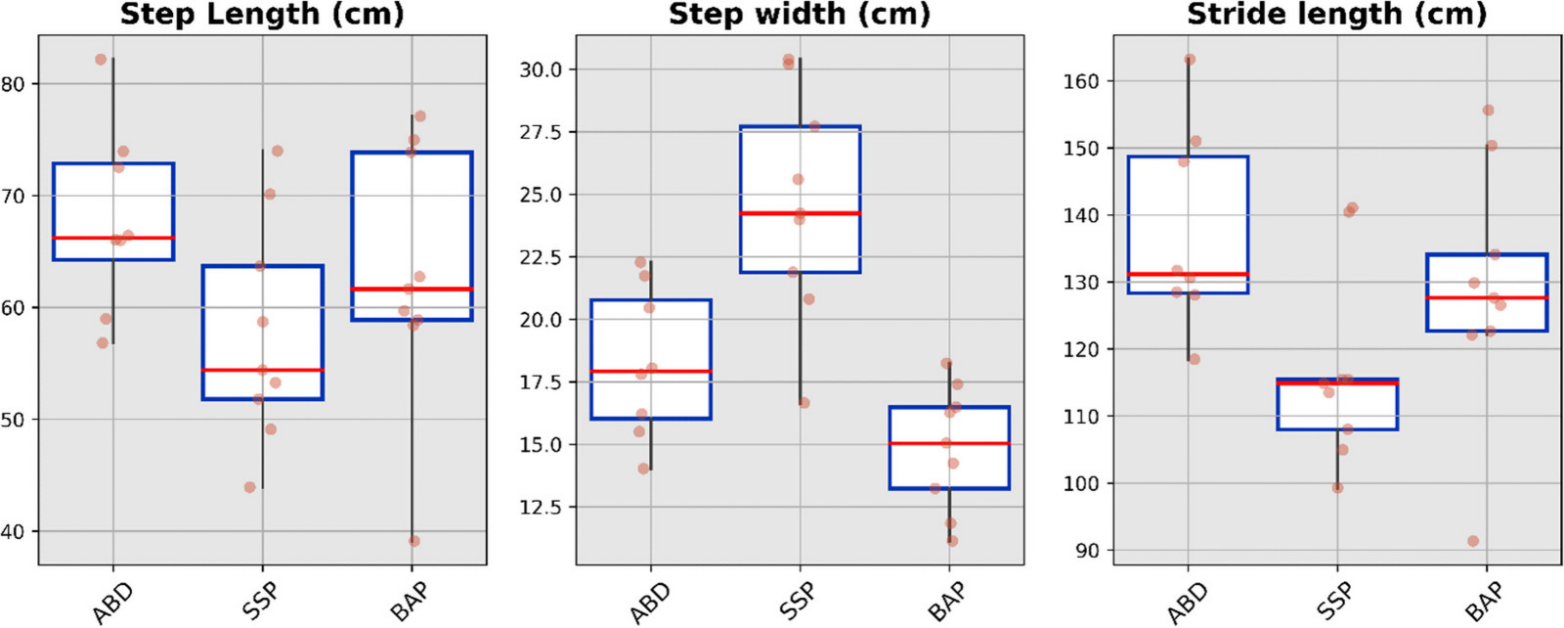
Boxplots showing median value, interquartile range and full range without outliers of the spatial parameters for ABD as well as individuals with transfemoral amputation fitted with SSP and BAP arms. ABD: able-bodied participants; SSP: socket-suspended prosthesis; BAP: bone-anchored prosthesis.

**Figure 5.**
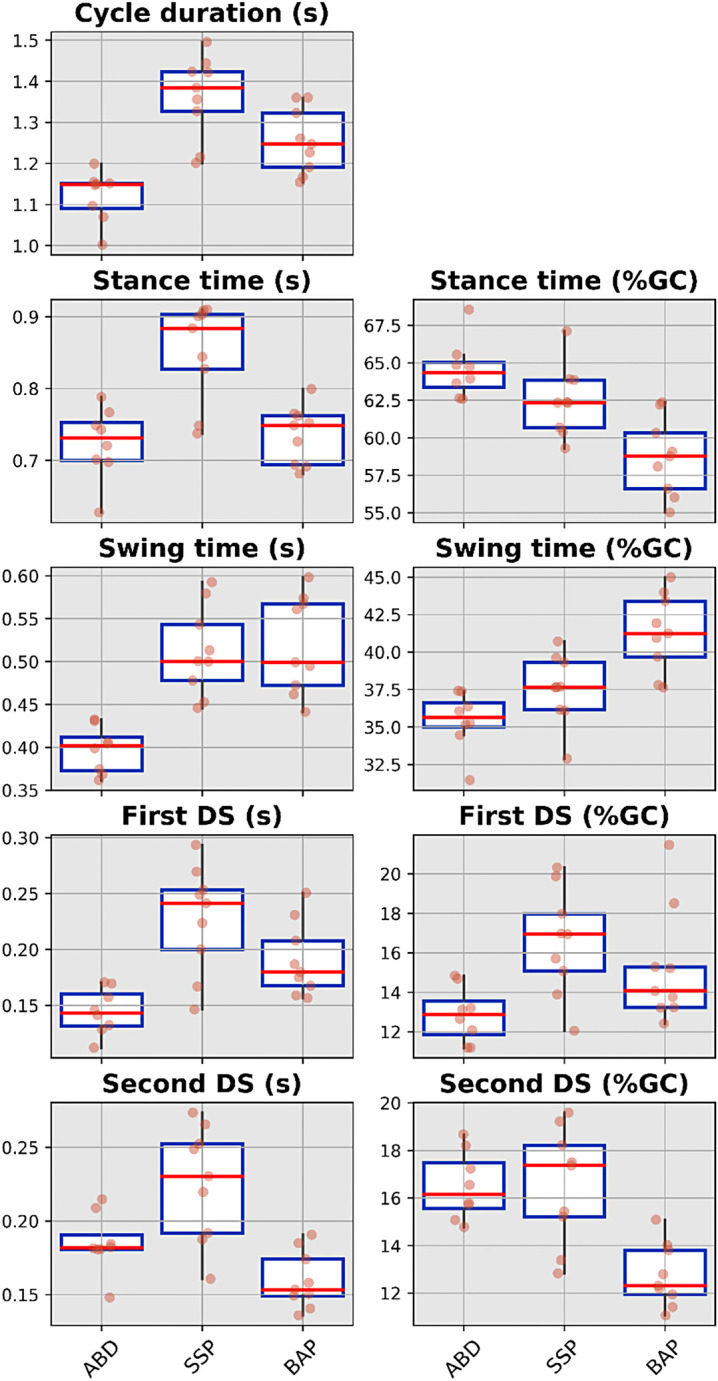
Boxplots showing median value, interquartile range and full range without outliers of the temporal parameters for ABD as well as individuals with transfemoral amputation fitted with SSP and BAP prostheses arms. ABD: able-bodied participants; SSP: socket-suspended prosthesis; BAP: bone-anchored prosthesis; %GC: percentage of gait cycle, DS: double support.

The median value, interquartile range and full range without outliers for the spatio-temporal, spatial and temporal parameters for each cohort were presented in Figures 3 to 5, respectively (Supplemental Tables S4 to S6). The cadence and speed of walking were 107 ± 6 steps/min and 1.23 ± 0.19 m/s for the ABD (Control), 88 ± 7 steps/min and 0.87 ± 0.17 m/s for the SSP (Control), and 96 ± 6 steps/min and 1.03 ± 0.17 m/s for BAP (Intervention) arms, respectively. The variability was high only for the first double support expressed in seconds extracted for the SSP arm out of the 42 gait parameters (e.g. 14 parameters × 3 arms).

Comparisons 1, 2 and 3 revealed eight significant differences (Table 3). For the intervention arm, the three significant differences were a longer swing phase for the BAP arm compared to the ABD arm, as well as a shorter step width and second double support in seconds for the BAP arm compared to the SPP arm.

**Table 3.** Comparisons of the gait parameters between ABD as well as individuals with transfemoral amputation fitted with SSP and BAP arms.

	ABD arm versus SSP arm		ABD arm versus BAP arm		BAP arm versus SSP arm	
	Mean ± SD	*p*-value	Mean ± SD	*p*-value	Mean ± SD	*p*-value
Spatio-temporal parameters						
Cadence (steps/min)	18.8 ± 9.3	0.000 *	11.3 ± 8.6	0.221	7.5 ± 9.1	0.349
Speed (m/s)	0.36 ± 0.26	0.007 *	0.20 ± 0.25	0.540	0.16 ± 0.24	0.438
Spatial parameters						
Step length (cm)	10.19 ± 12.88	0.240	4.93 ± 14.24	0.971	5.27 ± 15.27	0.693
Step width (cm)	−6.35 ± 5.40	0.189	3.38 ± 3.88	0.664	−9.73 ± 5.12	0.001 *
Stride length (cm)	20.44 ± 20.86	0.049	8.52 ± 23.70	0.930	11.92 ± 23.44	0.368
Temporal parameters						
Gait cycle (s)	−0.24 ± 0.12	0.000 *	−0.13 ± 0.10	0.221	−0.11 ± 0.13	0.349
Support phase (s)	−0.13 ± 0.08	0.098	−0.01 ± 0.06	1.000	−0.12 ± 0.08	0.113
Support phase normalized (%GC)	2 ± 3	0.880	6 ± 3	0.138	−4 ± 3	0.733
Swing phase (s)	−0.11 ± 0.06	0.005 *	−0.12 ± 0.06	0.005 *	0.01 ± 0.08	1.000
Swing phase normalized (%GC)	−2 ± 3	0.880	−6 ± 3	0.138	4 ± 3	0.733
First double support (s)	−0.08 ± 0.05	0.003 *	−0.05 ± 0.04	0.143	−0.04 ± 0.06	0.771
First double support normalized (%GC)	−4 ± 3	0.027	−2 ± 3	0.340	−1 ± 4	0.888
Second double support (s)	−0.04 ± 0.04	0.390	0.03 ± 0.03	0.752	−0.07 ± 0.04	0.009 *
Second double support normalized (%GC)	0 ± 3	1.000	4 ± 2	0.051	−4 ± 3	0.029

SD: one standard deviation, %GC: percentage of gait cycle, DS: double support; ABD: able-bodied participants; SSP: socket-suspended prosthesis; BAP: bone-anchored prosthesis.

*Significant difference (*p* < 0.017).

As presented in Table 4, Supplemental Figures S2 to S5, the ABD arm showed only 1 (2%) substantial correlation between height and second double support normalized as well as five (9%) moderate correlations including one between age and step length, four between height and support phase normalized, swing phase normalized, second double support in seconds and %GC, and one between mass and swing phase. The SSP arm showed no moderate and eight (14%) substantial correlations between mass, as well as body mass index and step width, support, swing and second double phases, normalized. The BAP arm showed no moderate or substantial correlations.

**Table 4.** Coefficient of determination (*r*^2^) and strength of correlation between the age, mass, BMI and gait parameters for of ABD as well as individuals with transfemoral amputation fitted with SSP and BAP arms (very weak: *r*^2^ ≤ 0.25, weak: 0.25 < *r*^2^ ≤ 0.50, moderate: 0.50 < *r*^2^ ≤ 0.75, substantial: *r*^2^ > 0.75).

	ABD arm				SSP arm				BAP arm			
	Age	Height	Mass	BMI	Age	Height	Mass	BMI	Age	Height	Mass	BMI
	(yrs)	(m)	(kg)	(kg/m^2^)	(yrs)	(m)	(kg)	(kg/m^2^)	(yrs)	(m)	(kg)	(kg/m^2^)
Spatio-temporal characteristics												
Cadence (steps/min)	0.02	0.04	0.12	0.41	0.43	0.09	0.01	0.00	0.41	0.06	0.10	0.11
Speed (m/s)	0.23	0.09	0.00	0.18	0.46	0.13	0.00	0.04	0.01	0.02	0.03	0.13
Spatial characteristics												
Step length (cm)	0.55	0.05	0.00	0.04	0.24	0.12	0.07	0.26	0.13	0.07	0.01	0.00
Step width (cm)	0.48	0.30	0.13	0.05	0.01	0.06	0.61	0.72	0.00	0.41	0.20	0.05
Stride length (cm)	0.36	0.09	0.02	0.05	0.41	0.15	0.01	0.10	0.03	0.06	0.00	0.07
Temporal characteristics												
Gait cycle (s)	0.02	0.03	0.13	0.38	0.42	0.10	0.03	0.00	0.41	0.05	0.09	0.10
Support phase (s)	0.01	0.24	0.00	0.31	0.18	0.01	0.06	0.10	0.38	0.04	0.09	0.08
Support phase normalized (%GC)	0.00	0.67	0.47	0.03	0.13	0.16	0.68	0.61	0.02	0.01	0.01	0.02
Swing phase (s)	0.02	0.27	0.74	0.15	0.49	0.25	0.40	0.28	0.20	0.03	0.04	0.06
Swing phase normalized (%GC)	0.00	0.67	0.47	0.03	0.13	0.16	0.68	0.62	0.02	0.01	0.01	0.02
First double support (s)	0.00	0.16	0.00	0.18	0.17	0.01	0.07	0.10	0.06	0.23	0.11	0.05
First double support normalized (%GC)	0.01	0.20	0.05	0.07	0.07	0.00	0.18	0.22	0.00	0.29	0.19	0.11
Second double support (s)	0.04	0.61	0.09	0.31	0.18	0.02	0.35	0.42	0.15	0.01	0.11	0.16
Second double support normalized (%GC)	0.03	0.76	0.33	0.11	0.05	0.10	0.57	0.60	0.00	0.00	0.04	0.07
Categories												
	(Nb)	(Nb)	(Nb)	(Nb)	(Nb)	(Nb)	(Nb)	(Nb)	(Nb)	(Nb)	(Nb)	(Nb)
Very weak	11	8	10	10	9	14	8	7	11	12	14	14
Weak	2	2	3	4	5	–	2	3	3	2	–	–
Moderate	1	3	1	–	–	–	4	4	–	–	–	–
Substantial	–	1	–	–	–	–	–	–	–	–	–	–
	(%)	(%)	(%)	(%)	(%)	(%)	(%)	(%)	(%)	(%)	(%)	(%)
Very weak	79	57	71	71	64	100	57	50	79	86	100	100
Weak	14	14	21	29	36	–	14	21	21	14	–	–
Moderate	7	21	7	–	–	–	29	29	–	–	–	–
Substantial	–	7	–	–	–	–	–	–	–	–	–	–

Nb: number, %GC: percentage of gait cycle, DS: double support; ABD: able-bodied participants; SSP: socket-suspended prosthesis; BAP: bone-anchored prosthesis; BMI: body mass index.

## Discussion

This study indicated that both ABD and BAP arms were comparable for all demographics as well as cadence and speed of walking, but the ABD arm showed a swing phase 31% shorter. Both BAP and SSP arms were comparable for all demographics as well as cadence and speed of walking, but the BAP arm showed a step width and duration of double support in seconds 65% and 41% shorter, respectively. The variability of gait parameters could only be partly explained by the variability of height and age for the ABD arm, the mass and body mass index for the SSP arm, and not explained by any of these potential confounders for the BAP arm.

One of the main limitations of this study described in Supplemental Table S7 related to the fitting of prosthetic components. Participants in BAP and SSP arms were fitted with a mismatch of knees and ankle/foot prosthetic component models although the ratio of knees with and without microprocessor-controlled was the same (Table 1).^16^ Furthermore, newer microprocessor-controlled knees and feet might be recommended nowadays.^13^ In principle, the design features of these advanced components should improve the walking ability (e.g. mechanically-powered push-off). However, the average cadence, durations of gait cycles and swing phase for our BAP arm were two steps/min (2%) faster, 0.1 m/s (7%) shorter and 4.3% GC (7%) longer than another cohort of participant fitted with state-of-the-art bone-anchored prosthesis recently presented by Frossard et al.^13^ In all cases, the fitting of newer microprocessor-controlled knees and feet are beneficial to reduce energy expenditure and gait stability (e.g. auto adaptive stance and swing phases, automatic stumble recovery).

The average cadence and duration of gait cycles for the ABD arm were eight steps/min (8%) slower and 0.03 m/s (3%) longer than those extracted from 10 benchmark studies presented by Frossard et al.,^11^ respectively. The differences for these parameters for the SSP arm was less than 1% compared to nine benchmark studies also presented by Frossard et al.^11^ Furthermore, conversion of these outcomes into the equivalent of a 6-min walk test showed that speed of walking and the distance walked could be 0.02 m/s (2%) faster and 7.11 m (2%) longer for BAP arm as well as 0.02 m/s (3%) slower and 8.76 m (3%) slower for the SSP arm compared to the benchmark data (Supplemental Table S8).^8–10^ Altogether, these results suggested that each cohort walked at a self-selected pace that was fairly comparable to benchmark data.

Overall, the three arms included participants with relatively comparable demographics and walking speeds. The comparisons of gait parameters showed only a few differences. These outcomes suggested that both attachments restored noticeably well the gait parameters, at a self-selected speed. In principle, the significant decrease in step width and duration of double support when fitted with bone-anchored prostheses could potentially indicate an improvement in gait stability. Studies suggested that osseoperception, an increase of hip muscle control, prosthetic knee and hip range of motion, the anatomical adduction angle of residual femur, and direct translation of ground reaction forces, altogether, might improve gait stability (Figure 1).^17–19^ Gailey et al.^20^ confirmed this assumption by showing that Activities Balance Confidence Scale increases significantly from 85.45 ± 12.34% to 94.31 ± 6.48% for participants with socket and bone-anchored prosthesis, respectively. Furthermore, Gibeaux et al.^21^ conducted a preliminary study showing the mediolateral margin of stability was wider, thereby, potentially safer for participants fitted with transfemoral bone-anchored prostheses than those with socket prostheses.^22^ The significant decrease in step width and duration of double support when fitted with bone-anchored prostheses could also potentially indicate a reduction of joint overuse. A decrease in step width and duration of second double support showed by the BAP arm might increase symmetry and reduce typical musculoskeletal overloading on sound hips and knee joints (e.g. osteoarthritis) and low back pain.^23^ Several studies showed that individuals with bone-anchored prostheses tend to put nearly half and over full body weight on the prosthetic limb during standing and ambulation, respectively.^12,13,24–27^ Finally, the extended duration of the swing phase showed by the BAP arm might be explained by the fitting of prosthetic knees with mechanisms unable to extend fast enough when walking at this self-selected speed.

Generalization of these outcomes must be considered carefully because the convenient sample size of each arm was approximately 11 participants smaller than typical studies but in bulk part of comparable studies focusing on lower limb kinetics.^9,10,28,29^ Nonetheless, the BAP arm represented a sensible portion of the population worldwide at the time of the study (15%). Benchmarking highlighted that outcomes were fairly comparable to previous studies, including those considering state-of-the-art components.^13^

In conclusion, this study confirmed that bone-anchored prostheses restored the walking ability of individuals with transfemoral amputation as well as conventional socket-suspended prostheses, at least for comparable self-selected speeds. Differences were only found for step width and duration of double support. These new benchmark gait parameters can be used to design future studies and develop algorithms for automated step identification capable of providing insights into the benefits of lower bone-anchored prostheses.

Altogether, this study is a worthwhile contribution towards closing gaps around the evidence of efficacy and safety of bone-anchored prostheses that will, hopefully, warrant the utmost favourable outcomes for a growing number of individuals living with limb loss opting for bionic solutions.

**Clinical Messages**

- The self-selected speed of walking of consumers fitted with transfemoral bone-anchored prostheses should be approximately 1.23 ± 0.19 m/s.
- The variability of gait parameters for consumers walking with transfemoral bone-anchored prostheses can be low.
- The fitting of transfemoral bone-anchored and socket-suspended prostheses restored equally well the gait parameters, at self-selected speed.

## Supplemental Material

sj-pdf-1-cre-10.1177_02692155231183779 - Supplemental material for Walking ability of individuals fitted with transfemoral bone-anchored prostheses: A comparative study of gait parameters Click here for additional data file. Supplemental material, sj-pdf-1-cre-10.1177_02692155231183779 for Walking ability of individuals fitted with transfemoral bone-anchored prostheses: A comparative study of gait parameters by Simone Ranaldi, Alexandre Naaim, Cristiano De Marchis, Thomas Robert, Raphael Dumas, Silvia Conforto and Laurent Frossard in Clinical Rehabilitation
