# Supplementary material for: Walking ability of individuals fitted with transfemoral bone-anchored prostheses: A comparative study of gait parameters
Source: Clin Rehabil. 2023 Jun 23;37(12):1670–83. doi: 10.1177/02692155231183779 (PMC10580681; doi:10.1177/02692155231183779)
Supplement: sj-pdf-1-cre-10.1177_02692155231183779 - Supplemental material for Walking ability of individuals fitted with transfemoral bone-anchored prostheses: A comparative study of gait parameters [file sj-pdf-1-cre-10.1177_02692155231183779.pdf]

Supplementary materials

The supplementary materials provide more information about the study design, definitions of gait parameters, seven-step process to extract and analyze gait parameters, descriptive statistics of the demographics and residuum information, descriptive statistics of gait parameters, regression analyses between four confounders and 14 gait parameters, list of limitations as well as benchmarking of 6 and 10-minute walk test data. Subsequent literature reviews and meta-analyses will be facilitated by additional information to be published in Data In Brief providing individual data for participants in each cohort.

Figure S1. Overview of the study design (arms, comparisons) and number of participants (N) in each of three arms recruited in Istituto nazionale Assicurazione Infortuni sul Lavoro (INAIL) Prosthetic Centre, Roma, Italy (Center A) and Department of Prosthetics and Orthotics of Sahlgrenska, University Hospital, Goteborg, Sweden (Center B). ABD arm: control arm with able-bodied participants, SSP arm: control arm with participants including transfemoral socket prostheses (SSP), BAP arm: intervention arm including participants with transfemoral bone-anchored prostheses

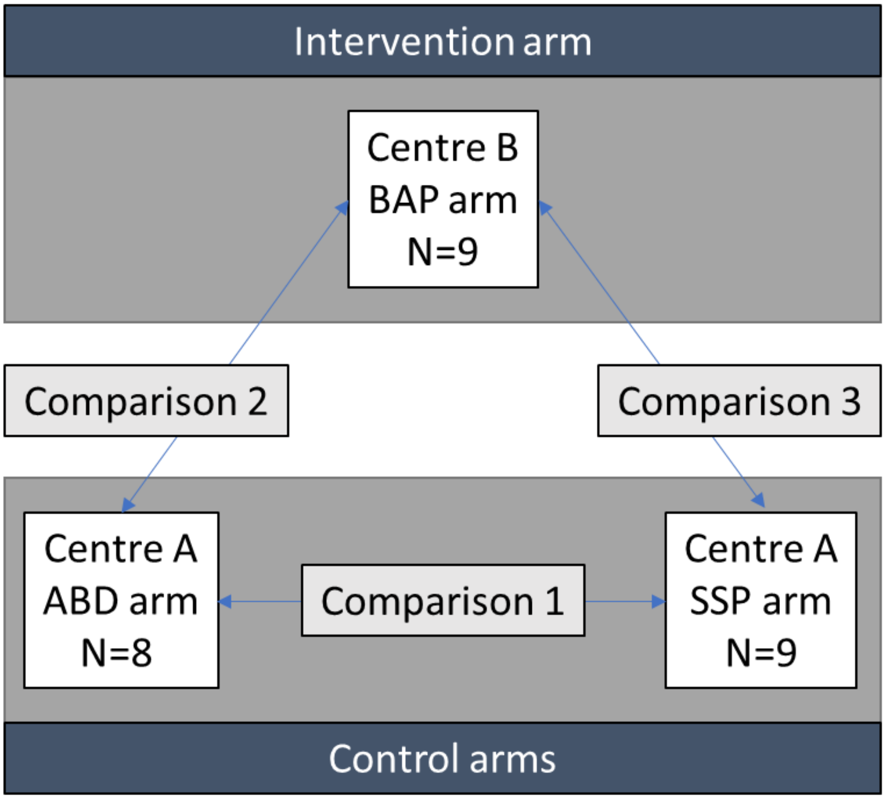

Table S1. Definitions of spatio-temporal, spatial and temporal gait parameters. [1] ***: variable discarded in this study due to discrepancies in positions of markers on the feet between arms. %GC: percentage of gait cycle**

Parameters	Definition
**Spatio-temporal parameters**	
Cadence (steps/min)	Number of steps per minute
Speed (m/s)	Distance travelled by the participant per seconds evaluated from the cadence and the stride length
**Spatial parameters**	
Step length (cm)	Anteroposterior distance between the right heel marker at right foot strike and left heel marker at left foot strike
Step width (cm)	Mediolateral distance between the right heel marker at right foot strike and left heel marker at left foot strike
Stride length (cm)	Longitudinal distance between heel marker at two consecutive foot strike
Minimum toe clearance (cm) *	Minimum vertical distance between the lowest point under the front part of the foot and the ground during mid-swing
**Temporal parameters**	
Gait cycle (s)	Duration of between two consecutive heel contact of same side
Support phase (s)	Duration of the stance phase in seconds
Support phase normalized (%GC)	Duration of the stance phase as a percentage of the gait cycle
Swing phase (s)	Duration of the swing phase in seconds
Swing phase normalized (%GC)	Duration of the swing phase as a percentage of the gait cycle
First double support (s)	Duration of the first double support phases in seconds
First double support normalized (%GC)	Duration of the first double support phases as a percentage of the gait cycle
Second double support (s)	Duration of the second double support phases in seconds
Second double support normalized (%GC)	Duration of the second double support phases as a percentage of the gait cycle

Table S2. Seven-step process applied to extract and analyse gait parameters using specifically written Matlab program (The MathWorks Inc., Massachusetts, United States of America). HC: Heel contact, TO: Toe-Off, DS: Double support

Step	Task	Description
1	Import raw data	The raw tridimensional kinematic and dynamic datasets from were imported.
2	Identify gait events	The tridimensional displacements of the makers placed nearby the heel and 5th metatarsal as well as the vertical ground reaction forces were used to identify heel-contacts and toe-offs of both prosthetic and sound limbs. For Experiments 1 and 2, gait cycles occurred between consecutive heel contacts. For Experiment 3, gait cycles were considered between consecutive heel contacts or toe-offs depending on the side of amputation and direction of walking. Gait cycles defined by toe-offs were temporally re-sequenced for consistency with the other datasets.
3	Harmonize coordinate systems	Displacements of the makers on both feet were measured in specific global coordinated system including a vertical, anteroposterior, and mediolateral axes in all three experiments. Some data were offset so that anteroposterior and vertical coordinates were nil at heel contact and during the central part of the swing phasis, respectively. All kinematic data were expressed in the same global coordinate system for comparison purposes.
4	Calculate gait parameters	Gait parameters of the prosthetic limb for SSP and BAP arms and single limb for ABD arm included two spatio-temporal (i.e., cadence, speed of walking), three spatial (i.e., step length, step width, stride length), and nine temporal (i.e., duration of gait cycle, support and swing phase and double supports) parameters (Table S8). The first and the second double support phases were defined as the transfer from the prosthetic heel contact to the sound toe-off and from the sound heel contacts to the prosthetic toe-off, respectively.
5	Normalize datasets	All spatial parameters were expressed in centimetres. All temporal parameters were expressed in seconds as well as percentage of the gait cycle that was time-normalized from 0 to 100.
6	Aggregate datasets	For any given parameter, all the trials for all the participants in each cohort we collated altogether. Datasets were characterised by their median value, their interquartile range and their full-range without outliers altogether represented as box-and-whisker plot. The variability of a dataset was determined using the percentage of variation (i.e., percentage of variation = absolute [(standard deviation/mean) x 100]). We considered that percentage of variation inferior or superior to 20% indicated a low and high variability, respectively.

7	Determine confounding effect of demographics	Possible confounding effect of age, height, mass, and body mass index on each of the 14 gait parameters were assessed considering at 56 correlations. We categorized coefficient of correlation (r) as very weak ($r \leq 0.25$), weak ($0.25 < r \leq 0.50$), moderate ($0.50 < r \leq 0.75$) or substantial ($r > 0.75$).

Table S3. Descriptive statistics of the demographics for able-bodied participants (ABD arm) as well as individuals fitted with transfemoral socket-suspended (SSP arm) and bone-anchored (BAP arm) prostheses. N: number participants, IQR: interquartile range, SD: one standard deviation, COV: coefficient of variation (COV=[standard deviation/mean]), CI: confidence interval, BMI: body mass index, SND: Sound thigh

	N	Median	IQR	Mean	SD	COV	95%CI-Lower	95%CI-Upper
**ABD arm**								
Age (Yrs)	8	52	7	54	9	0.17	47	60
Height (m)	8	1.75	0.09	1.75	0.07	0.04	1.70	1.80
Mass (kg)	8	74.50	7.75	76.25	6.69	0.09	71.61	80.89
BMI (kg/m^2)	8	25.189	1.938	24.959	1.958	0.08	23.602	26.316
**SSP arm**								
Age (Yrs)	9	62	7	59	10	0.17	52	65
Height (m)	9	1.70	0.10	1.73	0.07	0.04	1.68	1.78
Mass (kg)	9	78.00	7.00	80.33	16.42	0.20	69.60	91.06
BMI (kg/m^2)	9	26.600	3.800	26.767	4.486	0.17	23.836	29.697
**BAP arm**								
Age (Yrs)	9	49	13	51	13	0.26	42	59
Height (m)	9	1.80	0.05	1.78	0.09	0.05	1.72	1.84
Mass (kg)	9	90.00	19.10	87.32	16.15	0.18	76.78	97.87
BMI (kg/m^2)	9	26.735	4.651	26.136	3.649	0.14	23.753	28.520
Time since amputation (Yrs)	9	15	10	20	15	0.74	11	30
Time since BAP (Yrs)	7	5	3	6	4	0.68	3	9
Length of residuum (cm)	9	19.69	7.40	22.04	6.27	0.28	17.95	26.14
Length of residuum (%SND)	9	52	17	52	11	0.20	45	59

Table S4. Descriptive statistics of the 14 gait parameters for the able-bodied participants (ABD) arm. N: number participants, IQR: interquartile range, SD: one standard deviation, CI: confidence interval, COV: coefficient of variation (COV=[standard deviation/mean]), PV: percentage of variation (PV = absolute [(COV x 100]), H: high variability (PV>=20%), L: low variability (PV<20%), %GC: percentage of gait cycle

	N	Median	IQR	Mean	SD	95%CI-Lower	95%CI-Upper	COV	PV	
**Spatio-temporal parameters**										
Cadence (steps/min)	8	104	6	107	6	103	112	0.06	6	L
Speed (m/s)	8	1.17	0.23	1.23	0.19	1.10	1.37	0.15	15	L
**Spatial parameters**										
Step length (cm)	8	66.23	8.65	67.86	8.23	62.16	73.56	0.12	12	L
Step width (cm)	8	17.92	4.74	18.25	3.00	16.17	20.33	0.16	16	L
Stride length (cm)	8	131.15	20.36	137.43	14.97	127.06	147.80	0.11	11	L
**Temporal parameters**										
Gait cycle (s)	8	1.15	0.06	1.12	0.06	1.08	1.16	0.06	6	L
Support phase (s)	8	0.73	0.05	0.72	0.05	0.69	0.76	0.07	7	L
Support phase normalized (%GC)	8	64	2	65	2	63	66	0.03	3	L
Swing phase (s)	8	0.40	0.04	0.40	0.03	0.38	0.42	0.07	7	L
Swing phase normalized (%GC)	8	36	2	35	2	34	37	0.05	5	L
First double support (s)	8	0.14	0.03	0.14	0.02	0.13	0.16	0.14	14	L
First double support normalized (%GC)	8	13	2	13	1	12	14	0.11	11	L
Second double support (s)	8	0.18	0.01	0.19	0.02	0.17	0.20	0.11	11	L
Second double support normalized (%GC)	8	16	2	16	1	16	17	0.09	9	L

Table S5. Descriptive statistics of the 14 gait parameters for the individual with transfemoral socket-suspended prostheses (SSP arm). N: number participants, IQR: interquartile range, SD: one standard deviation, CI: confidence interval, COV: coefficient of variation (COV=[standard deviation/mean]), PV: percentage of variation (PV = absolute [(COV x 100]), H: high variability (PV>=20%), L: low variability (PV<20%), %GC: percentage of gait cycle

	N	Median	IQR	Mean	SD	95%CI-Lower	95%CI-Upper	COV	PV	
**Spatio-temporal parameters**										
Cadence (steps/min)	9	87	6	88	7	84	93	0.08	8	L
Speed (m/s)	9	0.81	0.06	0.87	0.17	0.76	0.98	0.20	20	L
**Spatial parameters**										
Step length (cm)	9	54.40	11.89	57.67	9.91	51.20	64.14	0.17	17	L
Step width (cm)	9	24.23	5.84	24.60	4.49	21.67	27.53	0.18	18	L
Stride length (cm)	9	114.89	7.43	116.99	14.54	107.49	126.49	0.12	12	L
**Temporal parameters**										
Gait cycle (s)	9	1.38	0.10	1.36	0.10	1.30	1.43	0.07	7	L
Support phase (s)	9	0.88	0.08	0.85	0.07	0.81	0.90	0.08	8	L
Support phase normalized (%GC)	9	62	3	62	2	61	64	0.04	4	L
Swing phase (s)	9	0.50	0.07	0.51	0.05	0.48	0.55	0.10	10	L
Swing phase normalized (%GC)	9	38	3	38	2	36	39	0.06	6	L
First double support (s)	9	0.24	0.05	0.23	0.05	0.20	0.26	0.21	21	H
First double support normalized (%GC)	9	17	3	17	3	15	18	0.16	16	L
Second double support (s)	9	0.23	0.06	0.23	0.04	0.20	0.25	0.17	17	L
Second double support normalized (%GC)	9	17	3	17	2	15	18	0.15	15	L

Table S6. Descriptive statistics of the 14 gait parameters for the individual with transfemoral bone-anchored prostheses (BAP arm). N: number participants, IQR: interquartile range, SD: one standard deviation, CI: confidence interval, COV: coefficient of variation (COV=[standard deviation/mean]), PV: percentage of variation (PV = absolute [(COV x 100]), H: high variability (PV>=20%), L: low variability (PV<20%), %GC: percentage of gait cycle, DS: double support

	N	Median	IQR	Mean	SD	95%CI-Lower	95%CI-Upper	COV	PV	
**Spatio-temporal parameters**										
Cadence (steps/min)	9	96	10	96	6	92	100	0.06	6	L
Speed (m/s)	9	1.01	0.13	1.03	0.17	0.92	1.14	0.16	16	L
**Spatial parameters**										
Step length (cm)	9	61.65	14.98	62.94	11.62	55.34	70.53	0.18	18	L
Step width (cm)	9	15.04	3.26	14.87	2.47	13.26	16.48	0.17	17	L
Stride length (cm)	9	127.62	11.43	128.91	18.38	116.90	140.92	0.14	14	L
**Temporal parameters**										
Gait cycle (s)	9	1.25	0.13	1.25	0.08	1.20	1.31	0.06	6	L
Support phase (s)	9	0.75	0.07	0.74	0.04	0.71	0.76	0.05	5	L
Support phase normalized (%GC)	9	59	4	59	3	57	60	0.04	4	L
Swing phase (s)	9	0.50	0.09	0.52	0.06	0.48	0.56	0.11	11	L
Swing phase normalized (%GC)	9	41	4	41	3	40	43	0.06	6	L
First DS (s)	9	0.18	0.04	0.19	0.03	0.17	0.21	0.17	17	L
First DS normalized (%GC)	9	14	2	15	3	13	17	0.19	19	L
Second DS (s)	9	0.15	0.02	0.16	0.02	0.15	0.17	0.12	12	L
Second DS normalized (%GC)	9	12	2	13	1	12	14	0.10	10	L

Figure S2. Regression analysis between the age and gait parameters for the able-bodied participants (ABD arm) as well as individuals fitted with transfemoral socket-suspended (SSP arm) and bone-anchored (BAP arm) prostheses including regression lines for moderate ($0.50 < r^2 \leq 0.75$) and substantial ($r^2 > 0.75$) coefficients of determination. %GC: percentage of gait cycle, DS: double support

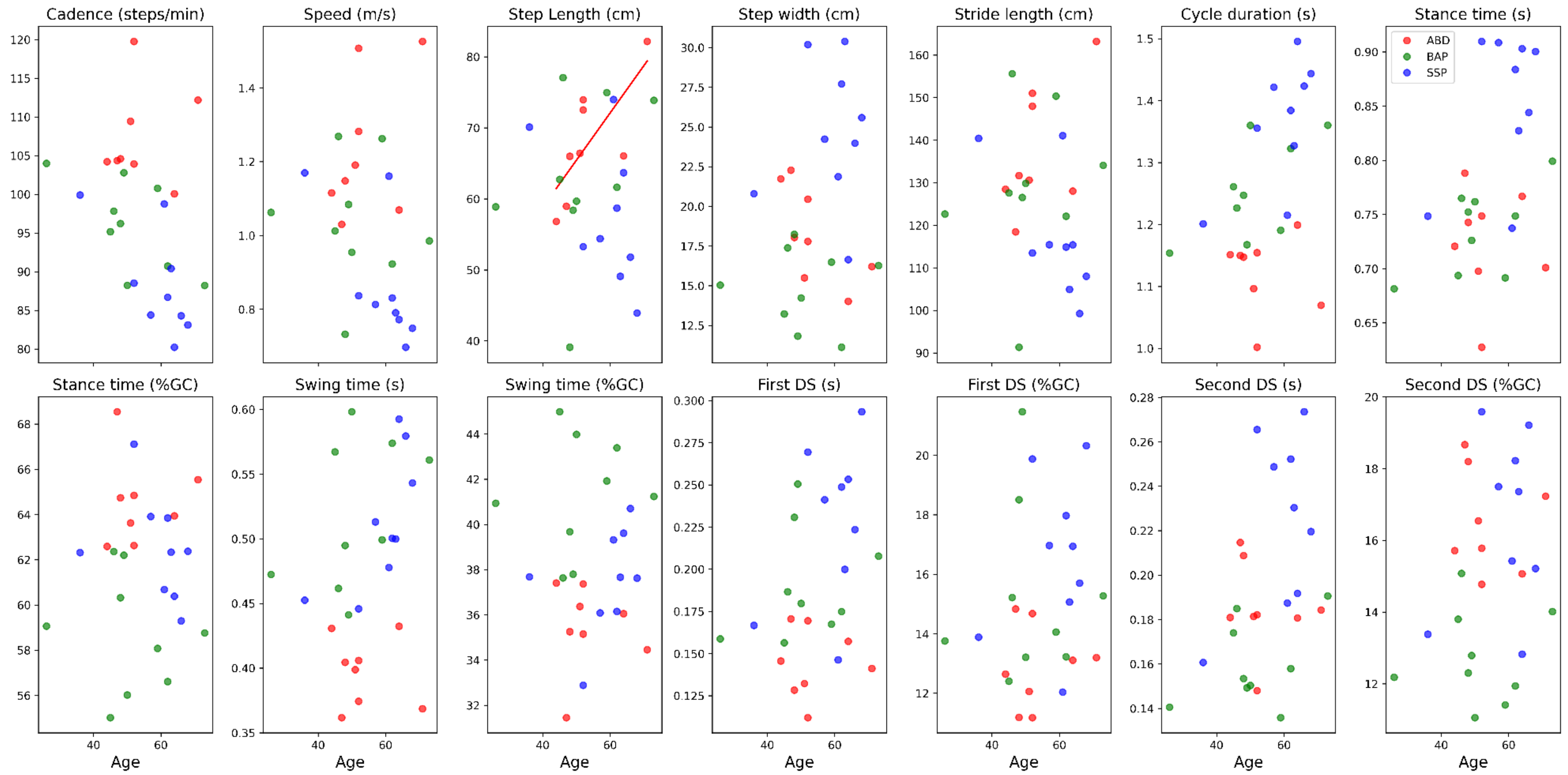

Figure S3. Regression analysis between the mass and gait parameters for cohorts of able-bodied participants (ABD arm) as well as individuals fitted with transfemoral socket-suspended (SSP arm) and bone-anchored (BAP arm) prostheses including regression lines for moderate ($0.50 < r^2 \leq 0.75$) and substantial ($r^2 > 0.75$) coefficients of determination. %GC: percentage of gait cycle, DS: double support

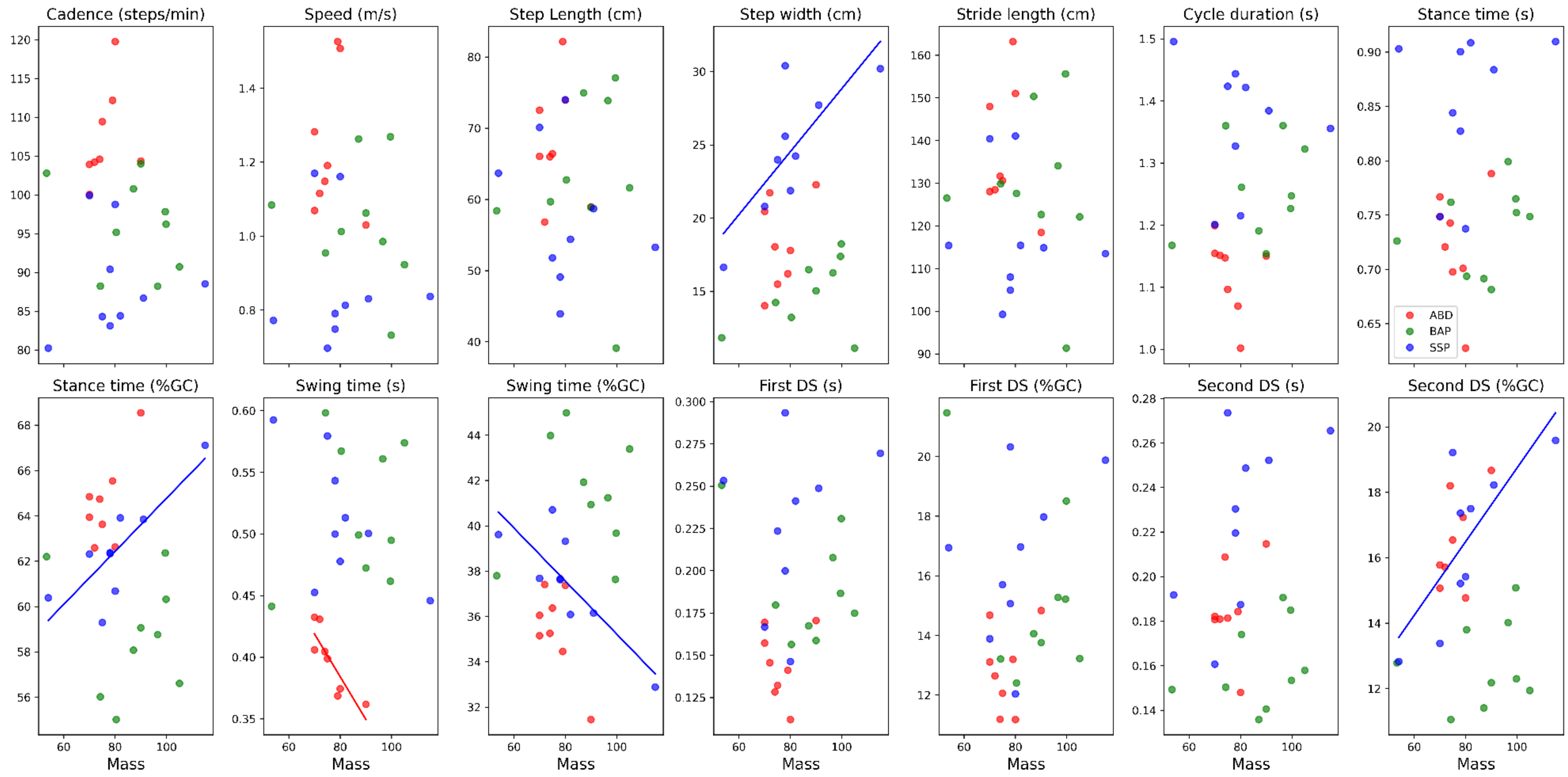

Figure S4. Regression analysis between the height and gait parameters for cohorts of able-bodied participants (ABD arm) as well as individuals fitted with transfemoral socket-suspended (SSP arm) and bone-anchored (BAP arm) prostheses including regression lines for moderate ($0.50 < r^2 \leq 0.75$) and substantial ($r^2 > 0.75$) coefficients of determination. %GC: percentage of gait cycle, DS: double support

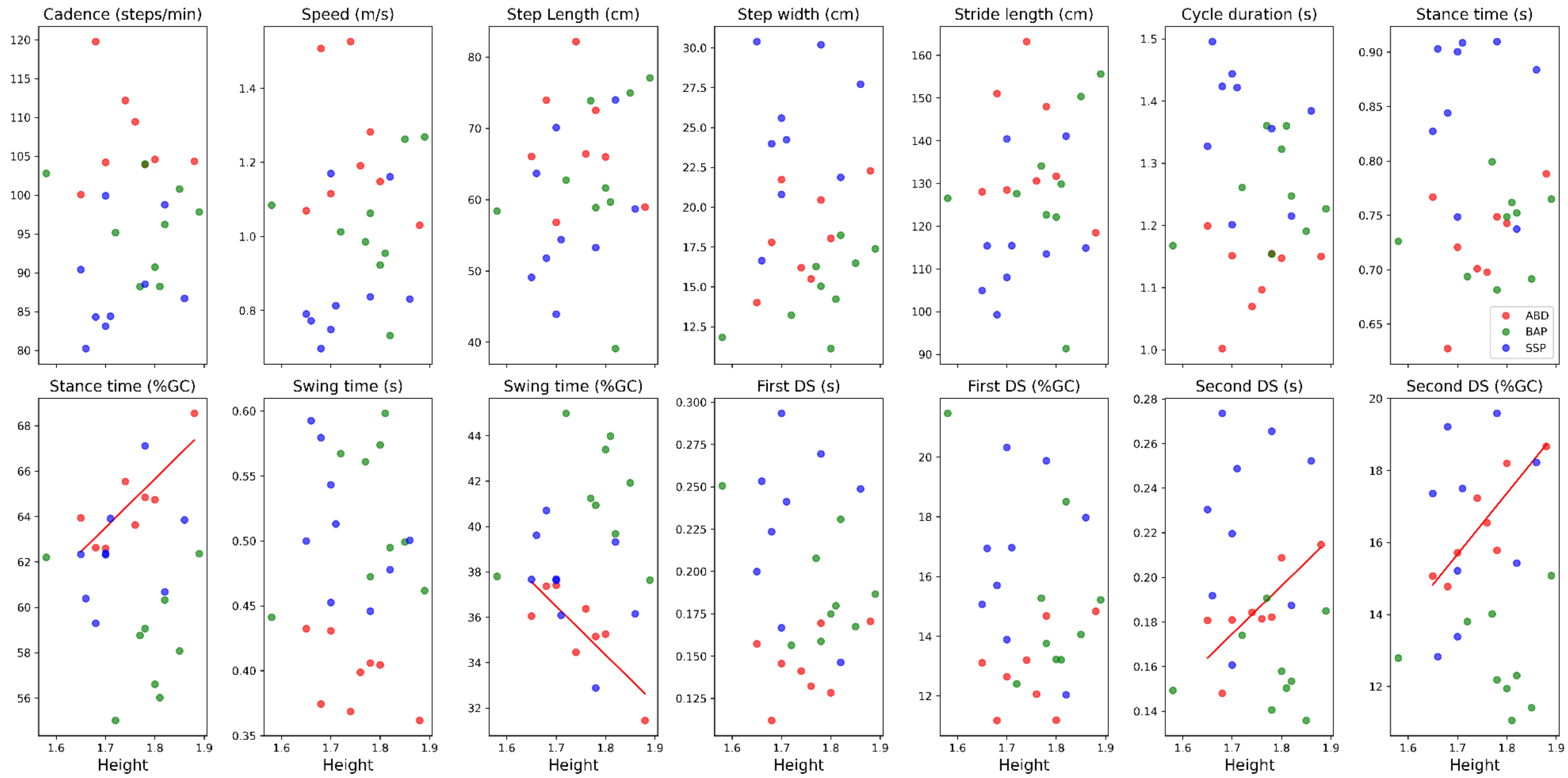

Figure S5. Regression analysis between the body mass index (BMI) and gait parameters for cohorts of able-bodied participants (ABD arm) as well as individuals fitted with transfemoral socket-suspended (SSP arm) and bone-anchored (BAP arm) prostheses including regression lines for moderate (0.50 < $r^2 \leq 0.75$) and substantial ($r^2 > 0.75$) coefficients of determination. %GC: percentage of gait cycle, DS: double support

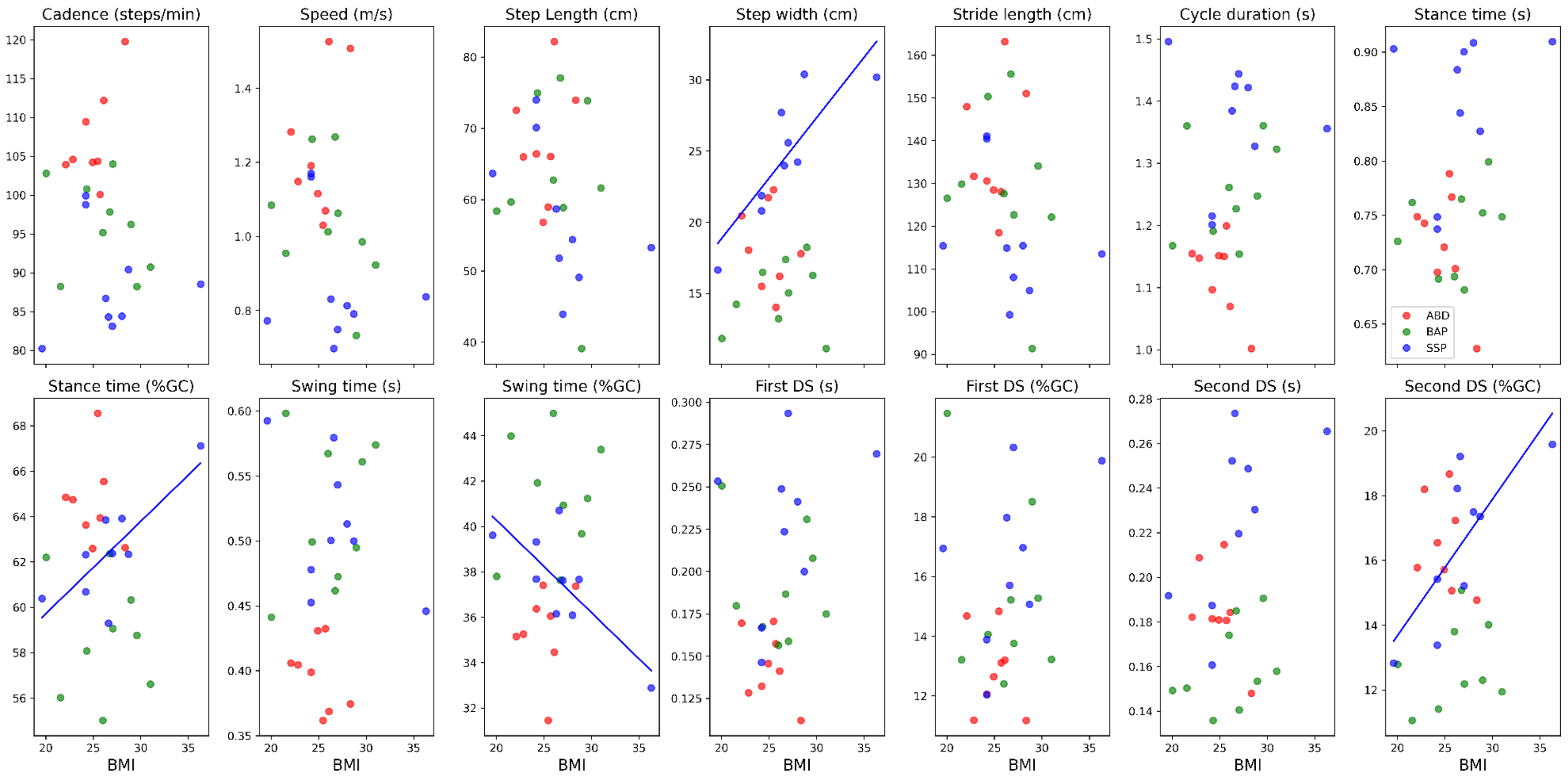

Table S7. Limitations of this retrospective cross-sectional study comparing cohorts of able-bodied participants (ABD arm) as well as individuals fitted with transfemoral socket-suspended (SSP arm) and bone-anchored (BAP arm) prostheses collected in difference centres.

Limitations	Description
Disparities in data collection protocols	• Gait parameters were extracted from raw kinematic data that might be confounded by variations between arms in:<br>○ Equipment (e.g., tridimensional motion capture systems).<br>○ Setups (e.g., volumes of calibration, size of walkways, force-plate arrangements, marker sets).<br>• Instructions given to participants (e.g., strategies to step over force-plates, number of gait cycles recorded).
Biased selection of participants with BAP	• The outcomes might be favourably biased by the recruitment the pain-free and active participants, because of the:<br>○ Inclusion criteria for the recruitment of BAP arm requiring that participants were able to walk 200 m independently with prosthesis for safety reasons.<br>○ Matching process where participants in SSP arm selected to match participants in BAP arm according to critical variables (i.e., age, height, weight, body mass index) without consideration for other unknown confounders (e.g., functional levels, cause of amputation, time since amputation, length of residuum).
Discrepancies in prosthetic fittings	• Mismatch of knees and ankle/foot prosthetic component models fitted between participants in BAP and SSP arms although the ratio of microprocessor-controlled and non-microprocessor-controlled was the same.
Selection of prosthetic components	• The gait parameters were obtained with components commonly prescribed at the time of the experiments.<br>• Newer microprocessor-controlled knees and feet might be recommended nowadays.
Missing confounding datasets	• SSP arm without consideration for other unknown confounders (e.g., functional levels, cause of amputation, time since amputation, length of residuum)were not initially recorded for Individual with transfemoral amputations with SSP.<br>• Difference in duration of acclimation with instrumented prosthesis between all arms.

Focused analysis	• Difference in inter-prosthetist variability in alignment between all arms.<br>• Foot clearance was discarded because of the discrepancies in positions of markers on the feet between the arms although minimum toe clearance is a critical spatial characteristic often associated with tripping probability and risk of falling.<br>• No consideration for minimal clinically important differences that individual participants would identify as important and meaningful to improve their mobility and health-related quality of live.

Table S8. Differences in outcomes 6-minute walk test and 10-minute walk test between this study and the average of three other studies also comparing individuals with transfemoral amputation fitted the bone-anchored and socket-suspended prostheses.[2-4] SD: one standard deviation, N: number of participants

		This study	Other studies	Van de Meent et al (2013)	Gailey et al (2017)	Sinclair et al (2022)	This study vs other studies
		Mean±SD	Mean±SD	Mean±SD	Mean±SD	Mean±SD	Difference (%)
		N=9	N=39	N=22	N=9	N=8	
**individuals fitted with transfemoral bone-anchored prostheses**							
Speed of walking	(m/s)	1.01±0.19	0.99±0.18	1.18±0.06	0.81±0.15	0.97±0.16	0.02 (2%)
6-minute walk test	(m)	362±69	355±66	423±21	292±54	350±58	7.11 (2%)
10-minute walk test	(m)	604±116	592±110	705±35	486±90	584±96	12.00 (2%)
**individuals fitted with transfemoral socket-suspended prostheses**							
Speed of walking	(m/s)	0.86±0.20	0.88±0.07	0.89±0.08	0.95±0.17	0.80±0.24	-0.02 (-3%)
6-minute walk test	(m)	308±73	317±27	321±28	342±61	289±88	-8.76 (-3%)
10-minute walk test	(m)	514±122	529±45	535±47	570±102	481±146	-14.33 (-3%)

1. Hollman JH, McDade EM and Petersen RC. Normative spatiotemporal gait parameters in older adults. *Gait & posture* 2011; 34: 111-118. Research Support, N.I.H., Extramural Research Support, Non-U.S. Gov't 20110429. DOI: 10.1016/j.gaitpost.2011.03.024.

2. Van de Meent H, Hopman MT and Frolke JP. Walking ability and quality of life in subjects with transfemoral amputation: a comparison of osseointegration with socket prostheses. *Arch Phys Med Rehabil* 2013; 94: 2174-2178. 20130614. DOI: 10.1016/j.apmr.2013.05.020.

3. Gailey R, Lucarevic J, Clemens S, et al. A Comparison of Prosthetic Mobility in Amputees with Osseointegration versus Traditional Amputation and Socket. *43rd Academy Annual Meeting & Scientific Symposium of the American Academy of Orthotists & Prosthetists*. 2017, p. FPTH14.

4. Sinclair S, Beck JP, Webster J, et al. The First FDA Approved Early Feasibility Study of a Novel Percutaneous Bone Anchored Prosthesis for Transfemoral Amputees: A Prospective 1-year Follow-up Cohort Study. *Arch Phys Med Rehabil* 2022; 103: 2092-2104. 20220729. DOI: 10.1016/j.apmr.2022.06.008.
